# Design of 3D-Printed Electronic Fiber Optic Sensor to Detect Rhodamine B Reagent: An Initiation to Potential Virus Detection

**DOI:** 10.3390/biomimetics7030094

**Published:** 2022-07-09

**Authors:** Ningyuan Guo, Jingwen Liu, Qing He, Rongyan Zhou, Haobo Yuan

**Affiliations:** 1 School of Electrical Engineering and Telecommunications, School of Engineering, University of New South Wales, Library Rd, Kensington, NSW 2033, Australia; z5342333@ad.unsw.edu.au; 2 UniSA STEM, University of South Australia, Mawson Lakes Blvd, Salisbury, SA 5095, Australia; liujy109@mymail.unisa.edu.au (J.L.); heyqy010@mymail.unisa.edu.au (Q.H.); 3 School of Marine Science and Technology, Northwestern Polytechnical University, Xi’an 710072, China; zhoury@mail.nwpu.edu.cn

**Keywords:** engineering design, rhodamine B, virus detection, antibody fluorescence reaction, COVID-19, ultraviolet light, product innovation, 3D printing, computer simulation

## Abstract

A fluorescence device based on ultraviolet light is proposed in this paper, which currently stands at the design stage with the eventual aim to potentially detect virus/antibody fluorescence reactions. The designed device is proposed to have the characteristics of high reflectivity, low power consumption, wide spectrum of light source, and proper silver coating. For fabrication and raising product quality, 3D printing technology and a sputtering test will be used. In this connection, this paper firstly introduces the design sources; then, the ideas of inventing fluorescence detection devices based on ultraviolet light, followed by the data analysis as well as discussing the results of computer simulations. The design process, materials, methods, and experiments are demonstrated following the reality work procedure. Instead of directly using a virus or antibodies for the experiment, at the current design stage, we focus on using this device to detect the rhodamine B reagent. Experiment shows that this reagent can be successfully detected. With this achievement, we logically believe that such type of an ultraviolet optical sensor, with further development and testing, may have the possible value to detect a single virus such as COVID-19, as well as other viruses or small molecules. Though there is long way to go to achieve such a goal, future works experimenting with the detection device on real virus or antibodies can take place more efficiently with a good foundation.

## 1. Introduction

With the COVID-19 pandemic in 2020, an increasing number of countries in the world have entered a closed state. Optical fiber detection technology is an effective and relatively fast detection technology for complex clinical laboratory detection [1,2]. Fluorescence detecting technology is used to rapidly detect the fluorescence response of virus antibodies, and to distinguish virus characteristics by measuring and recording its sensitivity values [3]. It senses signals through optical fiber conductors and presents the detection results and is used in the fields of medicine and biology. The goal of this research is to develop a small optical detection device with high sensitivity and high accuracy for virus detection. We used ultraviolet light as the light source and selected 12 UV-LEDs from the 255 nm–395 nm wavelength range. The average rated power is less than 150 mW, with the use of different low-power UV light sources to make a single low-power UV-LED driver. The UV-LED driver is made by using one diode (NPN, NMOS) and another diode as a conduction tube to form a switching circuit, so the operating current of each driver circuit can be matched with the UV-LED. The difficulty lies in the needs to match each set of UV-LEDs in different ways. Through calculation and circuit simulation, we successfully completed several sets of works for UV-LED simulation. By using Solidworks, we modelled a container and fabricated it with 3D printing technology, which can be used as the container for fluorescence detection experiments. This product is new and innovative. To obtain a better fluorescence detection effect, we also add a layer of silver coating to the test tube to ensure that the ultraviolet light source can be better reflected and can present us with a high-quality test effect. Silver is selected due to its higher reflectivity over other metals. After completing the coating experiment, the fluorescence detection experiment was conducted focusing on the detection of the rhodamine B reagent. Results are promising which fulfil designers’ original proposal. Though at current stage, real viruses are not experimented, it is predicted that this designed optical-fiber detection device could possibly be applicable for detecting viruses in a later stage with the improvement of the current design.

The current design idea was inspired via studying the previously developed detection methods that can be summarized as follows. The use of an optical fiber as a transmission signal to detect the pH characteristics of virus antibodies was conducted by Atias et al. [4]. This method is undoubtedly accurate, because it is serum-based and experimental. It mainly distinguishes patients by detecting their pH values, but it cannot do rapid detection. Optical biosensors are indispensable in today’s environmental analysis, food safety, biology, and medicine [5]. The fluorescence detection is popularly used for biosensors [6]. A fluorescent bioimmunoassay device embedded with silica nanoparticles was tested [7] that uses CdTe quantum dots (QD) as a signal. A similar device was also used to collect the laser-induced fluorescence (542 nm) into a photomultiplier tube (PMT) through glass fiber reflection [8]. Paper-based chips are manufactured by wax printing, which allows antibodies to be directly attached to the surface. CdTe quantum dots label silica nanoparticles on the signal antibodies and complete the detection. Studying the above documents, a virus detection sensor based on the fluorescence detection method can be an interesting future direction. The sensor is expected to be excited by a light source instead of other intermediaries [9]. The volume of design is also an important factor to be considered for convenience research purpose.

The next question comes with how the UV-LEDs can be used for Fluorescence detection. A fluorine-containing fiber optic biosensor was developed by using UV-LED Formaldehyde, methyl mercaptan, etc., which uses an ultraviolet LED as a fluorescence excitation source to measure ethanol [10]. In addition, a flexible and wearable enzyme glucose sensor can be used to monitor the glucose concentration. It is feasible to use a fiber optic sensor and an ultraviolet LED as a fluorescence excitation source to detect fluorescent proteins [11]. The virus has RNA, which means this method can be useful for fluorescence detection [12]. From another perspective, UV-LED can be efficient and has advantages. The miniaturization and low power consumption of the optical system are suitable for light sources [13]. For experimental light sources, UV LEDs are a very efficient choice [14]. Absorption detectors based on deep ultraviolet LEDs show excellent performance and potential for miniaturization for on-site analysis. The test shows that UV-based LEDs have excellent performance. Koshida et al. [13] described the fluorescence biosensing system with a UV-LED excitation for l-leucine detection. UV-LEDs can also be assembled into an optical system with a miniaturized and low-cost power consumption system [15]. The intensity of the fluorescent signal is determined by time; thus, to optimize the spectrum, the design of the PMEH-coated sensor is of importance [16,17]. A multiwavelength (250 to 355 nm) deep UV-LED detector was recently published by Kraiczek et al. [18,19]. for end-column detection. The detector performance was reported to be comparable to commercial detectors in the single wavelength mode but worse in the multiwavelength mode. Considering these, the low-power UV-LEDs excitation system can be composed of a 20 mA, 150 W low-power system, due to its higher economy. In this way, we will select this power range and operate the UV-LEDs system in the current range. In addition, since the intensity of a multiwavelength detector is weaker than that of a single-wavelength detector [20], to make the experimental effect more obvious, the single-wavelength detector is used and multiple UV-LEDs (using more combinations of UV-LEDs) between around 255 nm to 395 nm to form the circuit. In this work, we will produce a UV-LEDs container which is simplified and easily fabricable, with multiple holders and multiple openings.

In this study, the current detection focus is on the rhodamine B reagent for several reasons. Firstly, Rhodamine b acts as an excellent compound stain. It is easily soluble in water and emits fluorescence that can be easily detected under the irradiation of ultraviolet light [21]. Secondly, studies show that it can be used as a stain for most bacteria or small molecules [22]. It can be used alone or in combination with other stains. Thirdly, its labeling time is short, the required probe concentration low, the influence on bacteria small, and the labeling effect good. Fourthly, this study uses several UV-LEDs with different wavelengths to form a wide wavelength band, aiming to detect small molecules in the wavelength range from 240 nm to 400 nm. Experiments at the current stage show that rhodamine b can fluoresce and be detected by the sensor under UV light irradiation in this wavelength range. Finally, its neutral influence on virus or bacteria in the fiber guarantees the accuracy of detection [23,24]. Note that to eliminate bacteria before detection, the device can be rinsed using solutions, for example, with an SDS solution for a period followed by rinsing the device with solution such as PBST to remove potential bacteria and dried after each measurement [25].

## 2. Materials and Methods

### 2.1. Preliminary Methods

**Figure 1:**
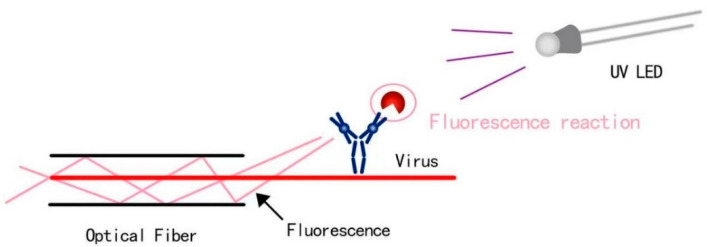
Detection principle in terms of fluorescence reaction and objects such as a virus.

Note that the optical fiber is a fiber made of glass or plastic that can be used for light transmission [26]. The transmission principle is “total reflection of light”. In this study, a UV-LED is emitting from the bottom direction. Rhodamine B is used a synthetic chemical and a dye that emits fluorescence (Figure 1). A real virus could be used in future design stages. The basic methods to support the foundation of our design chiefly include four aspects, which are light source, UV driver design, LED control by UNO, and the design of container and coating.

#### 2.1.1. Light Source

We firstly collected the working principle of triode and its design and the learning process of the triode analog circuit. The principle of a triode, such as how to control the triode as a switching circuit, and how to make triode conduction (voltage regulation or current condition of triode conduction), is analyzed strictly. We also calculated the basic triode circuit in physics, the resistance of the multi-channel LED circuit, and the required series resistance while matching the selected UV-LED-rated current.

#### 2.1.2. UV Driver Design

The circuit simulation software was used to create the resistance, UV-LED, transistor, power supply, and control terminal form 12 UV-LED driver circuits. Adding an ammeter (mA) to the circuit to simulate the circuit result was tested. Due to the existence of the transistor, the voltage drop (Vce) varies with temperature, and we adjusted both the UV-LED series resistance and the operating current of the target circuit which helps match the operating current of the UV-LED.

#### 2.1.3. LED Control by UNO

The single-chip microcomputer was used, and codes were written using Arduino. The goal is to make all 12 UV-LED Drivers controlled by one control source, ensuring that LEDs can be turned on and off regularly.

#### 2.1.4. Container and Coating Design

A stable container that can fix the UV-LED was developed. Computer modeling was conducted via Solidworks and the fabrication through 3D Printing technology. Considering it is necessary to enhance the reflectivity of the outer layer of wire, we also used a test tube with a special coating to perform a fluorescence detection experiment, where a sputtering test was conducted to test the coating’s stability.

### 2.2. The Method for Choosing the Light Source

From the literature, a UV-LED has a long bandwidth, high performance, low power consumption, and low price [27,28]. It is suitable as a light source and applicable for our aim. In this study, a UV-LED-based light source is applied with the decided wavelength range at about 200–400 nm. Note that the voltages of the UV-LEDs need to be similar with the rated small power.

#### 2.2.1. Selecting UV LEDs

In terms of voltage similarity, the output voltage is set at 3.5–6.3 V to ensure more convenient voltage adjustment after connection. In addition, the radiation intensity (power) of each UV-LED is set to be about 1–100 W to meet the requirements of low power consumption. For the wavelength of the selected UV-LED, the value is about 200–400 nm for convenience purpose, as most of the UV lamps on the market are of this range. Last, after screening at Digi-key, we determined the following UV-LEDs in compliance with the wavelength range (Table 1).

#### 2.2.2. Spectrogram Drawing and Data Analysis

**Figure 2:**
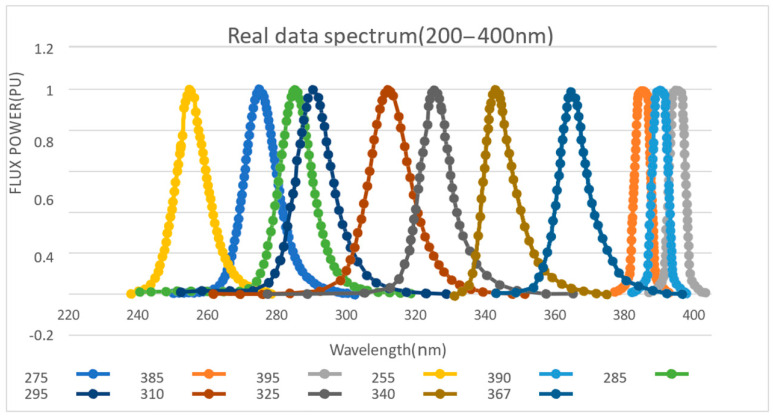
Light source spectrum.

Checking the data details of each UV-LED on the Digi-key official website, we drew the target spectrum simulation. By using the GetData Graph digitizer, the specific parameters of the image node are obtained, following which is the image output through Excel, as shown in Figure 2.

From the graph, 12 UV-LEDs basically include the spectral range from 200–400 nm, with a relatively large density. Since the three types of LEDs (UV3TZ Series) at 385 nm, 390 nm, and 395 nm are produced by BIVAR, their waveforms are similar and most concentrated. The effect of using this frequency for ultraviolet fluorescence with high probability is considered as the best option. Similarly, the 275–285 nm waveform area is also dense, meaning that it can be used as another priority for fluorescence detection experiments.

### 2.3. The Method for UV Driver Design

The above has explained how the light source was selected, and we identified 12 UV-LEDs (255 nm to 395 nm). To control these LEDs, we designed LED Drivers with different voltage drops and operating currents. This part discusses the design principles and simulations of LED Drivers. Due to the similarity of the design for each LED driver, here, we only selected two samples to illustrate the logic, process, and result of the design.

#### 2.3.1. The Use of Transistors

NPN structure and NMOS structure can be used as a switch and control circuit principle for design.

*Principle for NMOS circuit*. The advantage of using transistors is that small currents can be used to control large currents [29]. To avoid a high current, we used NMOS tubes. The reason is that the maximum through current of the NMOS may be greater than the maximum through current of the conventional NPN and that the conduction of the NMOS tube mainly depends on the voltage difference (threshold voltage) to determine whether the circuit is on or off. To avoid the influence of a high current on circuit control when using an NMOS tube, attention was paid to its Vgh ensuring the voltage drop was higher than that.

*Principle for NPN circuit.* For the UV-LEDs with a working current less than 100, an NPN tube is used. The NPN tube judges whether the circuit is open according to the current. This principle is based on the amplification effect of the transistor (making the base current multiplied by the diode’s amplification factor greater than the collector current to turn on the circuit). To be specific, the input voltage Vin controls the open and closed actions of the transistor switch. When the transistor is in the open state, the load current is blocked. On the contrary, when the transistor is in the closed state, the current can flow. In detail, when Vin is of low voltage, there is no current at the base and no current at the collector. As a result, the load connected to the collector has no current, which is equivalent to the opening of the switch. The triode is then better than the cut-off area. Similarly, when Vin is of high voltage, due to the base current flows, namely a larger amplified current flow through the collector, the load loop is turned on which is equivalent to the closure of the switch, and the triode works better in the saturation zone. For low-current UV-LEDs, the circuit can be turned on by setting Ib* β higher than Ic.

#### 2.3.2. Data Calculation and Circuit Simulation

The calculation process and simulation results of the two selected design cases are illustrated in the Supplementary Materials document (S1). This demonstrates that the logic and method of circuit design are reasonable, with desirable results obtained. Two sets of UV-LED Drivers using NPN tubes and NMOS tubes, respectively, are demonstrated. From the simulation, the green square shows that the voltage fulfils the proposed value range, and the red circle shows that the LED works properly. All other LED designs containing data calculation and circuit simulation follow the same procedure.

### 2.4. Design of Circuit Control

After completing the design of the circuit, the work of identifying the ultraviolet light source will be finalized. We proposed a concept called ‘Control’. This concept points out that to meet the requirements as ‘flexible, fast and accurate’ (FFA) detection, the ultraviolet light detection equipment in the project should be ‘controlled and controllable’ by the operator. In this way, the equipment can adjust the detection method, time and other parameters in different situations and targets. This makes the detection process relatively flexible and accurate. In addition, the remote control of the detector can be turned on and off at a specific time, and the time and frequency of the detector’s irradiation can be adjusted, which greatly optimizes the efficiency of the detector and makes the detection work easier.

#### 2.4.1. Design of Circuit Control

To meet the above requirements, we conducted several investigations and studies. The control method used should be able to connect remote equipment (such as a computer, etc.) with the circuit, and be able to control the circuit by issuing instructions. Finally, this control method can be continuously adjusted for the situation and purpose. We decided to use a single-chip device to control the circuit. A single chip microcomputer is a kind of an integrated circuit chip. It uses VLSI technology to integrate central processing unit CPU with data processing capabilities, random access memory RAM, read-only memory ROM, various I/O ports and interrupt systems, timers/counters, etc. Functions (may also include display drive circuits, pulse width modulation circuits, analog multiplexers, A/D converters, etc.) integrated into a small and complete microcomputer system on a silicon chip are widely used in the field of industrial control application. As a control system, a single-chip microcomputer can control the UV-LED circuit well.

**Figure 3:**
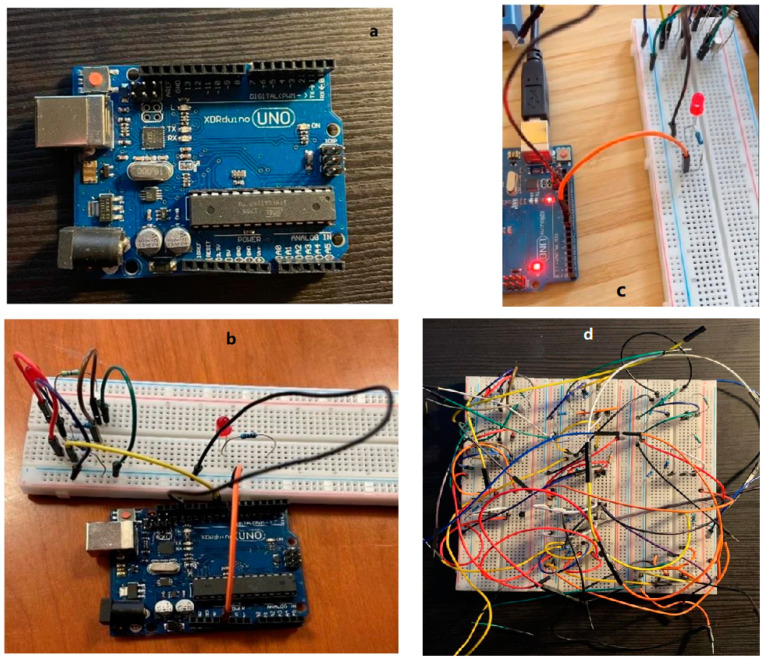
Arduino device used in this study (**a**), Arduino connected with UV-LED circuit (**b**), Arduino with LED On (**c**), fully assembly UV-LED Circuit (**d**).

We use the Arduino controller for the UV-LED circuit. The Arduino board is designed with various microprocessors and controllers. This circuit board is equipped with a set of digital and analog I/O pins, which can be connected to various expansion boards or breadboards and other circuits. These circuit boards have serial communication interfaces, including Universal Serial Bus (USB), and are also used to load programs from personal computers. Microcontrollers usually use the C/C++ programming language. The Arduino uno ^a^ (superscript ‘a’ corresponds with the part marked in same ‘a’ in Supplementary Materials) is used in this study; for the real product see Figure 3a, an open-source microcontroller board based on the Microchip ATmega328P microcontroller. The finalized electrical circuit alone can be seen in Figure 3d.

#### 2.4.2. Connecting Arduino with Circuit

To control the circuit, we first connected the circuit on the breadboard to the Arduino UNO device. As shown in Figure 3b, we use the extension wire to connect the circuit, and the other end of the wire is connected to the UNO development board. The resistor connected to the base is connected to the lead on the Arduino. Pin connection (PIN 9), the negative pole of the circuit is grounded (GND pin), and the positive pole of the circuit is connected to the 5.5 V pin, so that the device can provide power for the circuit (Figure 3c).

#### 2.4.3. Programming and Starting the Control Circuit

After completing the connection of the device to the circuit, we use the Arduino device to control the circuit. The method of control is to use the Arduino Software IDE, which uses a programming language such as C and C++ and provides common input/output Function. Programming information is obtained through the Wiring software library.

Completing the programming program and uploading the program to the Arduino development board, the circuit will be controlled according to the set program. Figure 3c shows the working condition of the UV-LED. The UV-LED is turned on and off with definite frequency according to the set program. This proves that the Arduino device can control the UV-LED circuit very well. For different needs, changing the programming program on the computer can realize the control of the UV-LED circuit. In this connection, the UV-LED can emit ultraviolet light periodically, controllably, and frequently, therefore, to achieve the desirable FFA detection. Information of example codes used in this programming is shown in the Supplementary Materials document.

### 2.5. Design of Fiber Optic Container (Inner Container)

**Figure 4:**
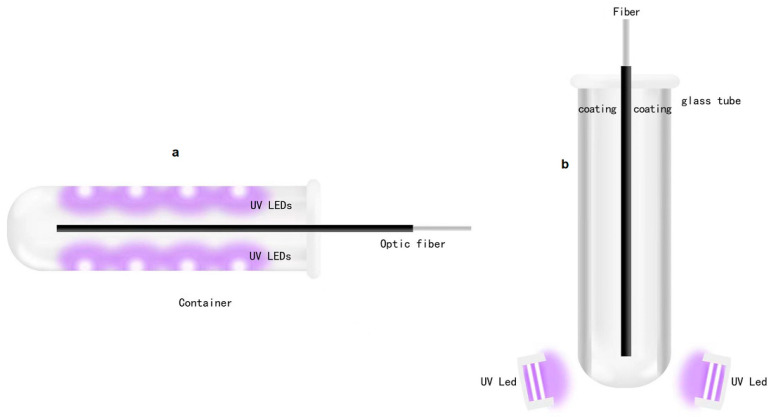
Preliminary design (**a**), sketch of coating (**b**).

After completing the circuit design, we obtained the proper light source for detecting use. At the same time, how to use a light source to detect optical fibers has become a new problem. How to combine optical fibers with UV-LEDs is also a concern of the design. A container is designed to hold the optical fiber and install UV-LEDs to irradiate the optical fiber. The initial plan sketch is shown in Figure 4.

Figure 4a shows the design of the semi-cylindrical container, insertion of the optical fiber to be detected into the container, inlay UV-LEDs for detection on the inner walls of both sides of the container, UV-LEDs which emit ultraviolet light to irradiate the optical fiber, and the detection of the light emitted from the surface of the optical fiber. Fluorescence is used to achieve the purpose of the experiment. However, in practice, we found that due to the small volume of UV-LEDs, it is difficult to mount them inside the container. At the same time, due to the low power of a single UV-LED, the brightness of the emitted ultraviolet light is not high, even if all the UV-LEDs embedded inside the container for irradiation also cannot obtain a light source of sufficient intensity, resulting in insufficient brightness of the fluorescent light generated on the optical fiber, which makes normal detection impossible. Therefore, this method is not feasible and a better option is needed.

Designing a new container is therefore necessary. To obtain a more concentrated light source, we decided to use a glass tube with special optical characteristics as the container for the optical fiber. The glass tube has special optical characteristics and does not refract light, thereby ensuring that ultraviolet light will not be interfered by the light of other wavelengths. UV-LEDs are not installed on both sides of the glass tube container, but placed on the bottom of the glass tube, emitting ultraviolet light from bottom to top. At the same time, it is necessary to ensure that the light source has sufficient intensity. A bright light source will increase the photocurrent used to calculate the absorbance (Sharma et al., 2015), making the fluorescence on the fiber surface more obvious and easier to detect. To achieve these goals, we prepared to deposit aluminum in the ANFF-South Australia (SA) facility (and also in another research group at FII) by sputter coating. The details of the method include a layer of silver or aluminum coating that is made on the inner wall of the glass test tube (it is found that both silver and aluminum have the characteristics of highly reflecting ultraviolet light through query data). The UV- LEDs at the bottom emit ultraviolet light to illuminate the inside of the glass tube, and the ultraviolet light will be combined with the coating After repeated reflections, the surface of the fiber is illuminated, so that a stronger light source can be obtained, and stronger fluorescence is formed on the surface of the light, which is more conducive to the detection of experiments. The sketch is shown in Figure 4b.

At present, there are two methods for making coatings, namely sputtering and Tollens’ test. Sputtering is performed in a vacuum system filled with inert gas. Through the action of a high-voltage electric field, the argon gas is ionized to generate an argon ion current and bombard the target. At the cathode, the sputtered target material atoms or molecules precipitate and accumulate on the glass to form a thin film. The advantage is that a thin film of high melting point material can be prepared at a lower temperature, and the original composition remains unchanged during the process of preparing the alloy and compound thin films. Tollens’ test is a chemical reaction in which a solution of a monovalent silver compound is reduced to metallic silver, and the resulting metallic silver adheres to the inner wall of the container to form a plating layer. We used two methods to make two coatings. After researching the data from Wikipedia/lbtek, the determined coating parameters are shown in Table 2. Using these parameters, coating is made in the ANFF-SA ^c^.

### 2.6. Design of Holder and External Container

After completing the UV-LED circuit design and the glass tube coating, a ‘container’ to assemble them to form a complete experimental device is needed and proposed to meet the following seven requirements:

- It can hold the glass tube, and the container is hollow.
- It supports the glass tube so that the glass tube does not contact the inner wall of the container to avoid wear of the coating and affect the brightness of the ultraviolet light.
- It needs to be airtight to avoid ultraviolet dispersion and coating wear.
- UV-LED can be installed on the surface of the container to illuminate the fiber.
- It needs to have a bracket so that the container can stand on the table for easy experimentation.
- Electronic components easy to install and remove.
- Low cost and easy to manufacture.

**Figure 5:**
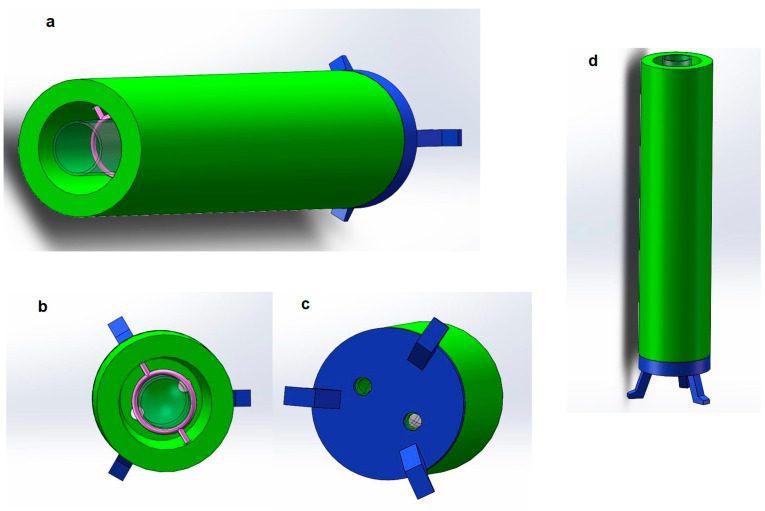
Container (Light chamber) (**a**), container top view (**b**), container bottom view (**c**), container standing on the desk (**d**).

To meet these requirements, we used 3D printing technology to manage and manufacture the container. 3D printing technology is low in cost, simple in production, and short in time, and through designing the geometrics, the product can be easily manufactured [30,31]. The materials used to make containers are mainly plastic, graphite, and carbon fiber [32,33]. These materials are strong enough, light in weight, and have good expandability. In this way, we firstly use Solidworks software to design the container model and then build the 3D model of the container. The 3D modeling diagram of the container is shown in Figure 5.

As shown in Figure 5a, the green part is the main part of the container, and the shape of the container is a hollow cylinder. Because the glass tube is also a cylinder, the inner hollow volume of the cylindrical container is very large, which can hold the glass tube well. In addition, the center of gravity is stable, and it can stand on the table well. Considering that the length of the optical fiber used in the experiment is about 100 mm to 200 mm, the height of the cylinder is designed to be 200 mm. The diameter of the container is 50 mm, and the inner diameter is 35 mm. There is enough space inside the container to prevent collision or friction between the glass tube and the inner wall of the container. The container will be made of a dense material, which is opaque to ensure that ultraviolet light will not escape.

At the same time, in order to ensure that the UV-LEDs can be installed on the container and are located at the bottom of the glass tube, there are two small openings with a diameter of about 1 mm on the other side of the container (see the lower bottom view for details) for inserting UV-LEDs. When the container is erected, the UV-LED is inserted into the small hole at the bottom of the container, towards the bottom of the container, so that the ultraviolet light can emit ultraviolet light from the bottom up, which meets the requirements, and does not sacrifice the tightness of the container to the greatest extent. In addition, according to the detection requirements, different UV-LEDs that can emit different wavelengths of ultraviolet light can be replaced as needed during the experiment. At the same time, it is easy to disassemble parts and modify, making the experiment process more flexible and having the advantages of modularization.

Figure 5b is a top view of the 3D modeling of the container. Through the top view, we can observe the inner part of the container more intuitively. The part of the figure in pink is a ring made of lightweight plastic, which is used to insert a glass tube. When fixing the glass tube, considering that the diameter of the glass tube used is about 20 mm and not more than 20 mm, the diameter of the ring is 20 mm. Two small holes recessed in the inner wall of the container are used to fix the ring on the inner wall of the container. Since the ring is made of plastic, its weight is very light and will not deform when suspended in the container.

The transparent part in the figure is the target glass tube, which is used to simulate the state of the glass tube inserted into it. It can be observed that after the glass tube is inserted into the container, the ring can fix the glass tube well, and the glass tube will not touch the inner wall of the container, and will not interfere with the assembly and detection of the optical fiber. In addition, it can be observed that the two small holes at the bottom of the container are located on both sides of the glass tube and are at an angle of 180 degrees to ensure that the intensity of the light emitted by the LED placed in it is uniform.

After completing the overall design of the container, to make the entire container stand on the desktop, a bracket for supporting the entire container must be designed. The above figure is the bottom view of the container. As shown, the blue part is a Bracket, it is used to support the entire container. It can be observed that there are also two small holes at the bottom of the bracket. Their position and size correspond to the small holes at the bottom of the container. UV-LEDs will pass through the bottom bracket and be fixed in the bottom position inside the container. The three supporting legs of the bracket are separated by 120 degrees and distributed in a triangle shape, which has good stability. The shape and size of the bracket are the same as those of the container. The two can be combined easily, and can also be easily disassembled, which is convenient for replacing electronic components and conducting experiments.

Figure 5d is the front view of the container and the stand, which simulates the actual state of the container standing on a plane. The coated glass tube and optical fiber will be inserted into the container from above and fixed by the ring. The UV-LEDs will be inserted into the bottom of the container from the small hole at the bottom of the container to irradiate. The bracket has a certain height from the plane, which is convenient for the bottom of the container. Placing more electronic instruments or circuits, UV-LEDs will use wires to connect to the remote circuit board, and the entire container design is completed. The parameters/dimension of the container are listed in Table 3.

## 3. Experiment and Result

### 3.1. Experiment Initiation

#### 3.1.1. UV-LEDs Circuit Assembling

After completing the design and simulation of the UV-LED circuit, the complete UV-LEDs circuit was assembled. We identified 12 UV-LEDs with different wavelengths that met the requirements (purchased through the Digi-key website). Other key electronic components were also obtained that make up the circuit, including: a large breadboard that can accommodate all independent UV-LED circuits, resistors of different values, NPN transistors, MOS tubes, and several wires. After all the components are obtained, they are assembled according to the circuit composition of the analog circuit diagram. The finalized UV-LED circuit after assembly is shown in Supplementary Materials.

Due to the lack of stock, two UV-LEDs cannot be purchased from the website, and there are no suitable substitutes, so we used 10 UV-LEDs. These ten separate UV-LEDs circuits are arranged in a manner from top to bottom and from left to right. Each circuit is composed of independent UV-LEDs, resistors of different values, and NPN transistors. The UV-LED parameters used are shown in Table 4.

#### 3.1.2. Container Fabrication via 3D Printing

**Figure 6:**
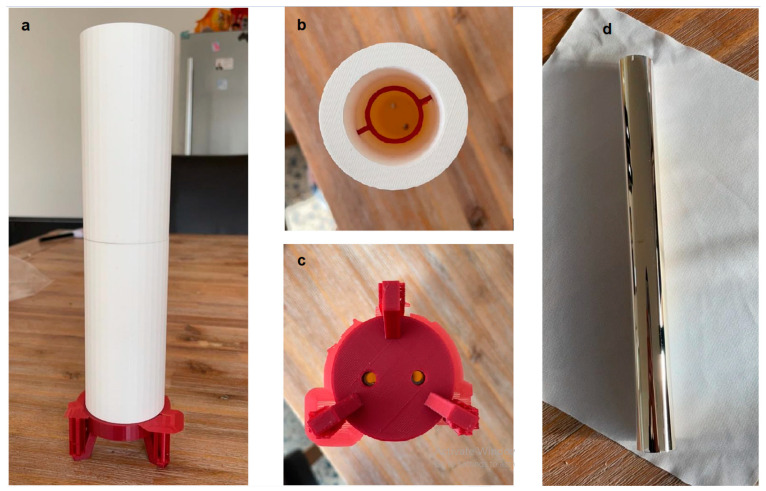
3D-printed container (**a**), top view (**b**), bottom view (**c**), the coated container after fabrication (**d**).

Based on the model created with Solidworks, the container was manufactured using 3D printing technology; the actual product shown in Figure 6a–c.

The main part of the container is made of a dense material, the red bracket at the bottom is made of plastic, and the ring inside the container is also made of light plastic and is embedded in the inner wall of the cylinder. The bottom of the container and the bracket have openings and correspond to each other. The container is lighter and meets with our proposed requirements.

#### 3.1.3. Container Coating

Following the manufacturing of the container is the coating process. Sputtering or Tollens’s Test was used to make the coating. A glass tube was obtained with special optical properties; it does not produce light refraction and can be used as a good carrier for making coatings. After that, we used sputtering to make the coating. The coating uses the determined parameters as the container and is made of aluminum and covers the outside of the glass tube, as shown in Figure 6d.

According to the above figure, it can be observed that the aluminum coating is evenly covered on the outside of the glass tube, has good reflectivity, and can meet the requirements of gathering ultraviolet light.

### 3.2. Fluorescence Detection Experiment

**Figure 7:**
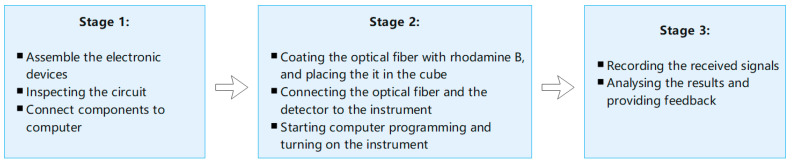
Illustration of the three experiment stages.

After completing the assembly and manufacturing of all components, it is necessary to verify the performance of the designed sensor device. Since the main purpose of this project is to design and develop an optical sensor, only preliminary experiments are carried out to detect whether the sensor can function normally according to the design purpose and detect fluorescent signals. This includes three stages, as illustrated in Figure 7.

#### 3.2.1. Experiment Stage 1

**Figure 8:**
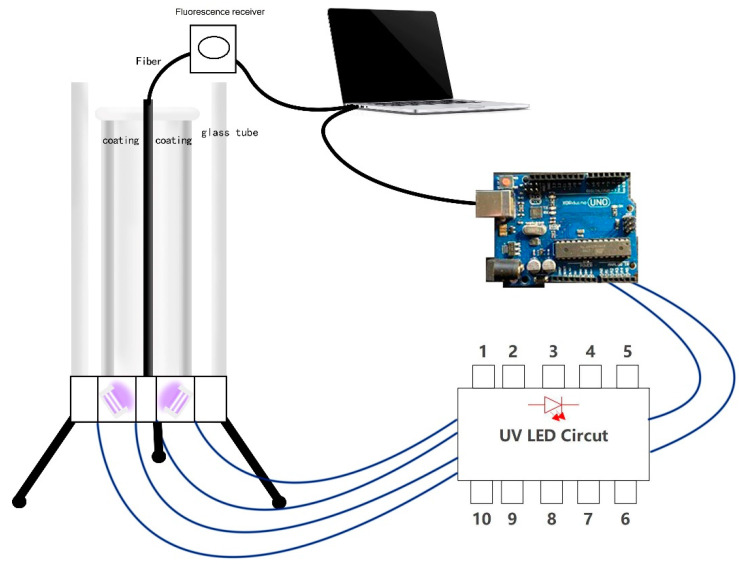
Sketch of sensor and illustration of connecting parts.

The experiment is carried out in the photonic optical laboratory at University of South Australia. First, all the sensor components are assembled, including the UV-LED circuit board, Arduino development board, container, and coated glass tube. The complete sketch of the optical sensor is shown in Figure 8.

Assemble the optical sensor as shown in the sketch. First, we did install UV- LEDs of different wavelengths according to the detection requirements, connect the UV-LED to the extension wire, insert the wire into the power circuit in the breadboard, and pass the UV-LED through the small hole at the bottom of the container. After that, we inserted it into the bottom of the container and fixed it. Because the rated current and rated voltage of the independent UV-LED circuit are relatively small (20–100 mA, 3–5 V), and the large independent power supply has the disadvantages of being too large and not easy to move.

**Figure 9:**
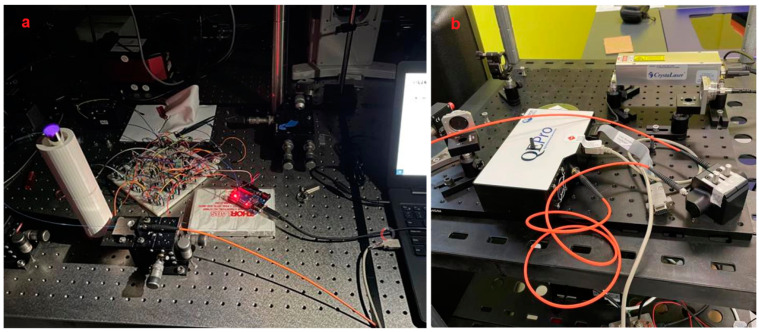
Real picture of the sensor taken under dark environment (**a**), the fluorescence receiver with optical fiber connected (**b**).

At the same time, the Arduino device comes with 3.3 V and the 5 V power pins, which can be connected to a computer directly as the power supply of the circuit, which is convenient for operation and testing. We connected the UV-LED circuit to the Arduino board, connected the positive pole to the 5 V power supply pin, the negative pole to the GND pin, and the transistor to the No. 9 pin for the control circuit. After that, we connected the Arduino to the computer using the USE cable. After completion, we inserted the coated glass tube into the container vertically from above, without contacting the inner wall of the container, so that it stands vertically inside the container. The complete optical sensor device is assembled. The physical figure is shown in Figure 9a,b.

#### 3.2.2. Experiment Stage 2

**Figure 10:**
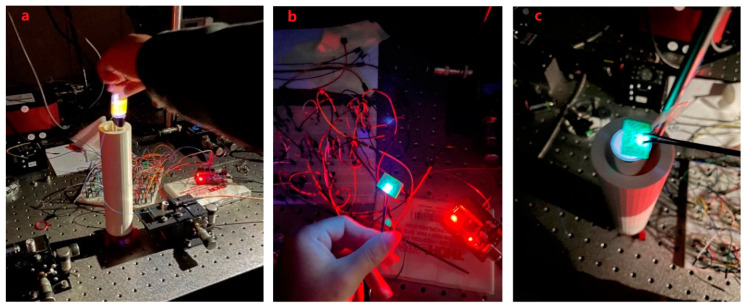
The yellow-light fluorescence of the Rhodamine B reagent under ultraviolet light (**a**), the designed LED (**b**), putting the UV-LED on top of the container in dark environment (**c**).

After assembling the sensor, we inserted the optical fiber to start the experiment. Because the purpose of the experiment is to check whether the sensor can work normally, the optical fiber does not include viruses or small molecular substances as carriers. To make the surface of the fiber fluorescent, we used the rhodamine B reagent instead of antibodies. Rhodamine B is a synthetic chemical and a dye that emits fluorescence, so it can be easily and cheaply detected by a fluorometer. Rhodamine dyes are also widely used in various biotechnologies [34]. Rhodamine B solution can be adsorbed by plastic, so it can be attached to the surface of the optical fiber, and it will fluoresce when irradiated by ultraviolet light [35,36]. Therefore, it can be used for preliminary experiments. Figure 10a shows the fluorescence of the Rhodamine B reagent under ultraviolet light.

The process includes to use the rhodamine B reagent to coat the surface of the optical fiber, connect the optical fiber to the measuring instrument, connect the measuring instrument to the computer with a USB cable, fix the light on the experimental table, and insert the end of the optical fiber stained with the rhodamine B reagent vertically from the top of the container in the glass tube.

After the sensor was ready, we did use the computer to open the Arduino coding program, upload the control code to the Arduino device, and then run it. As shown in Figure 10a, the UV-LED circuit is activated, the UV-LED emits light, and the ultraviolet light is irradiated into the light chamber from the bottom of the container. The figure also shows the experimental effect of the yellow color reflected light for detecting Rhodamine B. It can be observed from the figure that the inside of the glass tube is filled with ultraviolet light, which proves that the coating attached to the outside of the glass tube and ultraviolet light are generated. To achieve light reflection, the reflected light illuminates the entire optical fiber, so that the ultraviolet light is concentrated on the entire surface of the optical fiber, and the intensity of the ultraviolet light is enhanced as expected.

#### 3.2.3. Experiment Stage 3

We use optical fiber as the carrier for detecting the target. When a UV-LED is used to illuminate the virus vector (here, Rhodamine B reagent is used instead), fluorescence will be generated on the surface of the carrier, and the generated fluorescence will be received by the optical fiber, and the optical fiber will transmit the fluorescence quickly. The purpose of identifying the target virus can be achieved by observing the fluorescence. We open the Ocean-viewer software on the computer, the software can identify the signal on the optical fiber and output the results to the computer for observation and analysis. As shown in the next section, the waveform displayed on the observer can be observed, which proves that fluorescence has appeared on the surface of the optical fiber and the fluorescence signal has been successfully received.

In addition, by replacing UV-LEDs with different wavelengths to irradiate the optical fiber, different fluorescent signals can be obtained on the computer, and these fluorescent signals can be analyzed. At the same time, using Arduino programming software to write code can control the light source circuit and adjust the time and frequency of UV- LED light to meet the needs of the experiment. To help readers grasp a clear vision about the chips while emitting light, Figure 10b shows the designed LED and c when putting it on top of container.

### 3.3. Result Discussion

**Figure 11:**
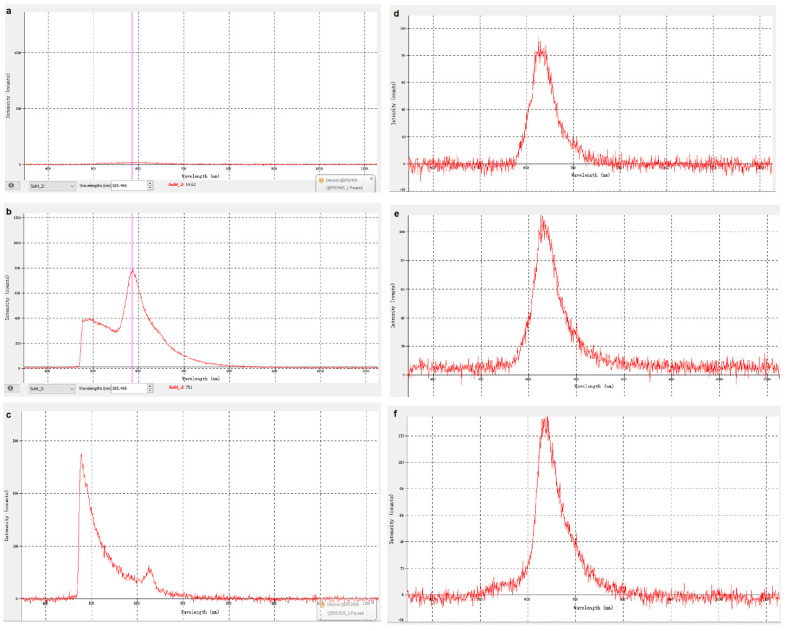
Spectrum without signal (**a**), fluorescent signal waveform 385 nm UV-LED without filter (**b**), 255 nm UV-LED-filtered (**c**), 275 nm (**d**), 310 nm (**e**), 325 nm (**f**).

The work and result of computer simulation will be illustrated in this section (observer settings d). UV-LEDs of different wavelengths are used to irradiate the optical fiber, and fluorescence of different wavelengths will be obtained on the surface of the optical fiber. We simulated the fluorescence emitted by different antigen carriers irradiated by ultraviolet light of a specific wavelength and observed the obtained fluorescence. the fluorescence signal curves were also analyzed on the observation software on the computer, see Figure 11.

Based on the above content, the experiment proved that the optical sensor works normally, can detect the fluorescence generated by the surface of the optical fiber and ultraviolet light, and can analyze the fluorescence signal. By changing to UV-LEDs of different wavelengths to irradiate the optical fiber, different substances can be identified (see the next section for specific principles and methods). It is worth mentioning that some UV-LEDs are not in the shape of a bulb (as shown in Figure 11). They are box-shaped and have a large PCB. They are large and cannot be inserted into the bottom of the container, so they can be installed directly on the bottom of the container. The top of the container directly irradiates the inside of the glass tube, which can also achieve the purpose of the experiment.

Figure 11b–f shows the fluorescence signal curves received by irradiating the fiber with a UV-LED with a wavelength of 255 nm, 275 nm, 310 nm, 325 nm, and a is the zero input as reference.

We can see that the large curve is the waveform of the fluorescent signal, and the small curve is the waveform of the background light of ultraviolet light. The wavelength and intensity of the fluorescence signal can be attained by observing the waveform of the fluorescence signal. To eliminate irrelevant variables, we used a filter to filter and adjust the ultraviolet light background signal. After that, we continued to irradiate the fiber with different wavelengths. The waveform diagram obtained is shown in Figure 11c, which can be compared with Figure 11b.

It can be observed that the fluorescence curve shapes are similar, and that the wavelength and intensity of the fluorescent signal generated by UV-LEDs of different wavelengths irradiating the fiber are different. Basically, with increasing the wavelength of UV- LED, the maximum intensity would increase, but remain at a hundred to thousand level. The peak area represents the definite number of wavelengths that LED occurs.

In actual experiments, the type and characteristics of the virus/antigen-carrier can be judged according to the wavelength and intensity of the fluorescent signal generated. This will help achieve the purpose of rapid, real-time, and convenient detection of small particles, which might include viruses.

The curve has jagged fluctuations. This might be caused by the insufficient brightness of the UV-LED used and the unstable fluorescent signal received. The power provided by the 5 V pin is still insufficient to operate the light source circuit under certain circumstances. The intensity of the ultraviolet light generated by the three UV-LEDs is too weak, which makes it impossible to receive a sufficient fluorescent signal.

In the subsequent improvement, a power supply with a higher rated power should be used to ensure that the UV light intensity generated by the UV-LED is greater. In addition, because the light source circuit is built on a breadboard, each circuit is too tight, and when installing different UV-LEDs for testing, it will be interfered by the components and wires of other circuits, which affects the efficiency of the experiment to a certain extent, and the circuit is not simple and beautiful. The light source circuit should be optimized in the future.

## 4. Conclusions and Future Recommendation

A fluorescence electrical device based on ultraviolet light is proposed in this paper, which aims to eventually detect virus antibody fluorescence reaction. Instead of directly using virus or antibodies for the experiment, at current stage, we chiefly focus on using this device to detect the rhodamine B reagent. The experiment shows positive results.

Taking the full picture of virus-detection into consideration, we have undertaken and completed the first part of work, especially in setting up the experimental devices, designing electronics, and fabricating the detector. This work chiefly contains the design of electronic components and the fabrication of a 3D-printed container and conducts the simulation test. Although the current computer analysis is limited, there is still an indication that this research direction is valuable. This result provides us with confidence to predict that such type of ultraviolet optical sensor will potentially be able to detect small molecules. For the future step of detecting a single virus such as COVID-19, the device could be applicable with further improvements.

On the other hand, such an easily set-up device, laboratory-invented, being cost-effective, with most resources flexibly available in daily life, could have potential to be applied in reality. An advanced electrical detection device with sophisticated and tailored control of LEDs can be inspirated through this study as the basic logic of design remains the same.

There are limitations and obstacles for us to undertake this research; for example, the extremely difficult scenario at the current time to perform experiments on in vitro virus applications. Therefore, we had to use the alternative for testing the design. Future works in terms of taking a real virus test, with the assistance of biomedical researchers, is necessary, which helps improve the current design and make the original proposal of this study come true. In this way, attaching a real virus in fiber becomes a concern, which can be achieved through immersing the optical fiber in an aqueous solution containing the virus, or directly applying the virus reagent on the surface of the optical fiber, and then inserting the optical fiber into the container.

In terms of future works, several aspects can be considered as valuable. Firstly, in the current study, only one LED works inside the container during the testing process, which means that we need to predetermine the light properties of the detected object to identify the proper LED to test and verify such an object. The efficiency of this can be improved by developing an integrated device with a full set of LEDs inside, so different objects to be tested could stimulate different LEDs, which helps researchers identify the detected object easily in a much more effective way. Currently, this work is undertaken by the authors’ group. Secondly, questions come how to deal with bacterial and other elements such as water contaminants during the detecting process. This work is important as bacterial and other contaminants could also trigger the light fluorescence, which could result in negative or misleading detection results. How to effectively distract objects such as a virus and purify both the detecting environment and the device itself therefore becomes a concern for future biotechnical researchers.

## Figures and Tables

**Table 1:** Filtered UV-LEDs overview.

Wavelength	Part Number	Price	Voltage	Power
395 nm	492-1321-ND	AU2.980	3.4 V	15 mW
390 nm	492-1511-ND	AU3.450	3.4 V	15 mW
385 nm	VAOL-5GUV8T4-ND	AU2.050	3.0 V	65 mW
367 nm	VLU1610-365-135TR-ND	AU1.391	3.3 V	19 mW
340 nm	1125-1370-ND	AU84.790	4.1 V	55 mW
325 nm	1125-1268-ND	AU196.500	5 V	0.8 mW
310 nm	2112-CUD1GF1ATR- ND	AU9.408	5.5 V	1.2 mW
295 nm	1125-1368-ND	AU80.070	6 V	1.5 mW
285 nm	2112-TUD8MF18-ND	AU31.500	6 V	10 mW
275 nm	2112-CUD7GF1BTR-ND	AU14.847	6 V	11.5 mW
255 nm	2112-CUD5GF1BTR-ND	AU182.566	7.5 V	3.5 mW

All UV-LEDs can be found through the Part Number, which can be found on Digi-key’s official website (www.digi-key.com, 6 February 2021). Each UV-LED is a mini-shaped optical LED and can be used for optical experiments. All Spectral properties are available in the official Datasheet.

**Table 2:** The Parameters of Coating.

Specification	Parameters
Production method	Sputtering &Tollens’ test
Thickness	5 mm
Wavelength range	250–450 nm
Reflectivity	Ravg > 89%
Typical energy density limit	0.5 J/cm^2^ @ 355 nm, 10 ns
Homogeneity	yes
Material	UV plus hard aluminum film or silver coating

**Table 3:** The Parameters of the 3D-designed container.

	Height	Outside Diameter	Inside Diameter
Container	200 mm	50 mm	35 mm
Bracket	35 mm	50 mm	/
Circle	/	/	20 mm

**Table 4:** The Parameters of 10 UV-LEDs.

Location	Part Name	Wavelength	Current	Voltage
1	VAOL-5GUV8T4	385 nm	30 mA	3.5 V
2	MTSM340UV	340 nm	500 mA	4.3 V
3	TUD8MF1B	285 nm	150 mA	6 V
4	MTSM310UV	310 nm	30 mA	6.2 V
5	CUD5GF1B	255 nm	100 mA	7.5 V
6	MTSM295UV	295 nm	30 mA	6.2 V
7	CUD7QF1A	275 nm	20 mA	6 V
8	VLMU1610	367 nm	20 mA	3.5 V
9	MTE325H21	325 nm	20 mA	5 V
10	CUD1GF1A	310 nm	30 mA	5.5 V

## Data Availability

Not applicable.

## Supplementary Materials

The following supporting information can be downloaded at: https://www.mdpi.com/article/10.3390/biomimetics7030094/s1, Figure S1: Sample codes of Blink program of Arduino; Figure S2: Two simulation cases, the 285 nm LED Driver, a, the 390NM UV LED, b.
