# Supplementary material for: Design of 3D-Printed Electronic Fiber Optic Sensor to Detect Rhodamine B Reagent: An Initiation to Potential Virus Detection
Source: Biomimetics (Basel). 2022 Jul 9;7(3):94. doi: 10.3390/biomimetics7030094 (PMC9326570; doi:10.3390/biomimetics7030094)
Supplement: Supplementary file 1 [file biomimetics-07-00094-s001.zip › biomimetics-1798738-supplementary.pdf]

Supplementary Material

S1:

[a] *The development board is equipped with digital and analog input/output (I/O) pin groups. Connect to various expansion boards (shield) and other circuits. The development board has 14 digital I/O pins (six with PWM output function), 6 analog I/O pins, and can be programmed with Arduino IDE (Integrated Development Environment) via a Type-B USB cable. It can be powered by a USB cable or an external 9-volt battery. In addition, this development board has 3.3V and 5V voltage pins, which can be used as a temporary power supply for the UV LED circuit. It has 13 external pins that can be connected to all UV LED circuits, and it also has a GND pin.*

[b] To write suitable execution programs according to the detection requirements and conditions. After compiling and linking with GNU toolchain, Arduino Software IDE provides a program "avrdude" to convert executable files to be able to burn and write Enter the firmware of the Arduino hardware, and after the program is programmed into the Arduino hardware, the device can control the operation of the circuit according to the program. At present, the need is to control the switch of the circuit and the irradiation frequency of the UV LED. Two steps are included. Use the USB cable to connect the Arduino UNO to the computer and use the Arduino Software IDE for programming on the computer. The Blink program of Arduino can meet the requirements. The program example is as follows, Figure S1.

```
int LED_PIN=9;

void setup () {                // Initialize the subroutine, only execute once at the beginning of
the program
   pinMode (LED_PIN, OUTPUT);     // Enable Pin9 with digital output
}

void loop () {                 // loop subroutine, repeatedly executed
   digitalWrite (LED_PIN, HIGH);  // open LED
   delay (1000);               // Wait for one second. Delay contains a value of 1000, which
represents a delay of 1000 milliseconds, which is one second.
   digitalWrite (LED_PIN, LOW);   // close LED
   delay (1000);               // wait for one second
}                           // End of loop subroutine
```

Figure S1. Sample codes of Blink program of Arduino

[c] *ANFF-SA*

https://www.anff-sa.com

The Australian National Fabrication Facility (ANFF) is a research infrastructure facility established under the National Collaborative Research Infrastructure Strategy.

[d] *The observer settings*

Date: Thu Jun 03 15:47:38 ACST 2021
User: Z
Spectrometer: QEP02405 Trigger mode: 4
Integration Time (sec): 2.000000E-1 Scans to average: 1
Electric dark correction enabled: true Nonlinearity correction enabled: false Boxcar width: 0
X-axis mode: Wavelengths
Number of Pixels in Spectrum: 1044

S2:

Two simulation samples, Figure S2, the 285Nm LED Driver, a, the 390NM UV LED, b.

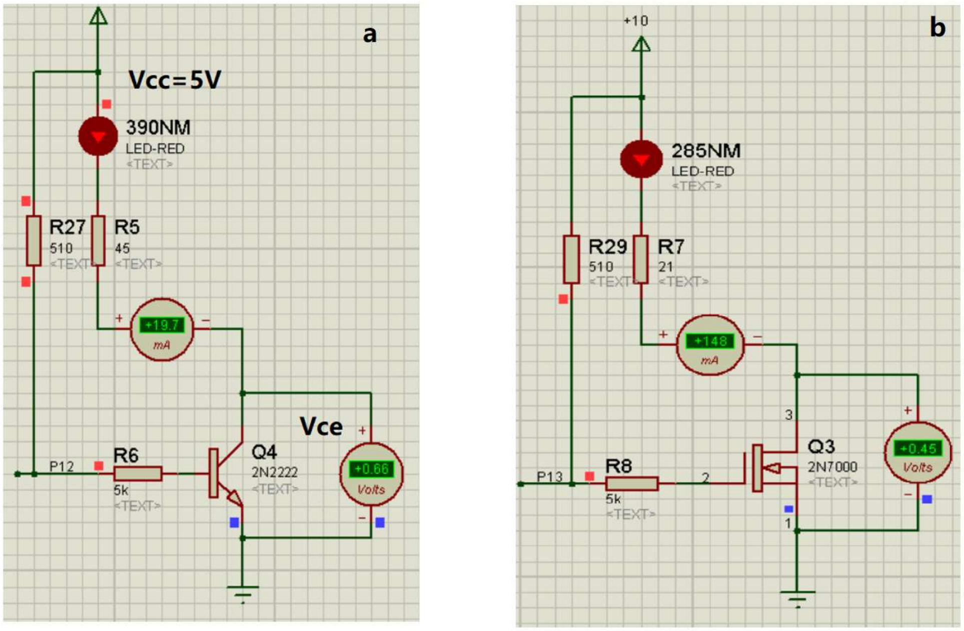

Figure S2. Two simulation cases, the 285Nm LED Driver, a, the 390NM UV LED, b.

The details about calculating the circuits are illustrated below:

<u>*Calculating the NPN circuit*</u>. Figure 3b shows the design of a 390NM UV LED driver. This circuit uses NPN tube. The parameters of the 395NM LED are 3.4v voltage drop and 20mA working current. To protect the circuit and to ensure that the working current of the circuit is synchronized with the 390nm UV LED, we added a resistor R5. Due to the increase in resistance and to ensure that the voltage of 390NM in the circuit is 3.4v, Vcc is set to 5V, so Vcc-V_{390nm} = 5-3.4 = 1.6v. The NPN transistor in the circuit will also have a voltage drop Vce, so the size of Vce is assumed to be 0.7v (silicon material). Therefore, the final voltage divided by the resistor R5 is Vr=1.6-0.7=0.8v. Since the operating current of the LED is 20mA, the approximate range of its resistance is 0.8/0.02= 40Ω.

According to the properties of the UV LED and transistor, it is necessary to connect a suitable resistor to obtain a suitable Vce. After adjustment, the measured Vce is close to 0.7V under the condition of R5=45Ω and Ic=19.7mA. The ammeter Ic = 19.7mA, which is close to the ideal operating current of the 390NM UV LED at 20mA. This proves the success of the circuit design. R27 and R6 are two larger variable resistors. The purpose is to control the size of the basic current so

that it can be multiplied by a magnification greater than Ic (20mA). In this design, all NPN drivers adopt the 2N2222 model, and the magnification is adjustable between 100 and 300. The saturation current collector voltage and base voltage of the UV LED in this design are all within the tolerance range of this NPN model tube.

Calculating the NMOS circuit. Take 285NM LED driver as an example, figure 3a. Since its working current is 150mA and the voltage drop is 6V, we designed a control circuit based on NMOS tube (Vcc = 10V). Since NMOS relies on the threshold voltage to determine whether the circuit can be fully turned on, we only need to ensure the G point voltage (represented as the voltage on node 2 in Figure 3a) and S voltage (represented as the voltage in Figure 3a, node 1 in Figure 3b). If the voltage difference Vgs of the NMOS (2N7000) tube is greater than the threshold voltage value of the NMOS (2N7000) tube used, it can be turned on. In the legend, the voltage of node 2 is 10V and the voltage of node 1 is 0 (ground), so its Vgs = 10V, the maximum Vgs of 2N7000 in normal mode is 3V and its maximum input voltage is 2V. Both the two poles of the three poles and the maximum input voltage are greater than 10V, Therefore, the NMOS tube can be fully turned on.

For R29 and R8 in Figure 3, the values are not unique, with 510Ω and 5KΩ selected. For the series voltage divider R7 of the UV LED, the calculation method is the same as that of the NPN structure circuit. It is worth noting that the voltage drop through the NMOS tube varies greatly in actual simulation, and it can be expected to set the voltage drop to 1V. Therefore, the calculated value of R7 is approximately: R7 = (10V (Vcc)-6V-1V) / 150 = 20Ω. After running the test, the voltage drop of the NMOS tube is close to 0.5V, and R7 to 21Ω. After operation, the working current is 148mA, which is close to the working current of 150mA. This proves in this case the circuit design is successful.
